# News Items

**Published:** 1872-01-01

**Authors:** 


### News Items.

In Vienna during the winter semester of the academical year 1870-71, there were 1,653 students of medicine, and during the past summer semester there were 1.460.

Prof. W. W. Dawson has been appointed to succeed the late Dr. Blackman in the chair of surgery at the Medical College of Ohio.

MEDICAL COLLEGES AND GRADUATES IN THE UNITED STATES.—Dr. Toner's statistics for the Department of the Interior show the number of Medical Colleges in the United States to be,

| | |
|---|---|
| Regular | 60 |
| Pharmacy | 16 |
| Dental | 8 |
| Homœopathic | 8 |
| Eclectic | 8 |
| Botanic | 2 |

Forty-eight of the colleges have furnished statistics for 1870, which show 4,989 matriculates, 1,500 graduates, 77 ad Eundem, and 15 honorary degrees.—*Buffalo Medical and Surgical Journal.*

THE British Medical Journal gives the total number of students registered at the London hospitals during the present season as 1,468. New entries, 468.

MISS PUTNAM, a young American who has been following for some years the course in l'Ecole de Medicine, at Paris, recently submitted her graduating Thesis to the faculty. The President of the Board of Examiners found it deserving of the highest note—"*extremement satisfait*"—a mark rarely given for a Thesis. She also received the highest mark at each of her examinations. Miss Putnam is the first woman who obtained admission to l'Ecole de Medicine, but not the first who has graduated, as a Miss Garrett took a year's course and received her degree some time ago.

UNDER the new (Prussian) Faculty at the University of Strasbourg there have been 11 examinations for degrees in the scholastic year 1870-71, against 1,014 in the scholastic year 1869-70.

CHOLERA.—In spite of the cold weather the epidemic still holds its own in Russia and Galicia. In some villages of the latter it has broken out afresh.

OPIUM IN CROUP.—The use of opiates in the treatment of true croup is warmly commended by Dr. J. S. Seaton in the *American Practitioner.* Repeated doses of Dover's powder are given until the distressing symptoms are abated and an emetic (ipecac) is required.

